# Hydrogen sulfide repairs testicular damage induced by heat stress in rats

**DOI:** 10.1002/2211-5463.70010

**Published:** 2025-03-05

**Authors:** Xinyu Guo, Yupeng Yin, Susu Guo, Xiaoyan Chen, Suyan Xie, Shiqin Chen, Yan Huang

**Affiliations:** ^1^ Center of Reproductive Medicine The General Hospital of Southern Theater Command Guangzhou China; ^2^ The First Clinical Medical College Southern Medical University Guangzhou China; ^3^ Department of Obstetrics and Gynecology General Hospital of Southern Theatre Command Guangzhou China

**Keywords:** H_2_S, heat stress, inflammation, oxidative stress, testis

## Abstract

Heat stress can cause testicular damage and affect male fertility. The gasotransmitter hydrogen sulfide (H_2_S) is involved in various physiological and pathophysiological processes, with antioxidant and anti‐inflammation effects. The aim of this study was to investigate whether H_2_S could reduce the testicular damage caused by heat stress. In this study, we constructed a rat heat‐stress model and treated it with H_2_S. Histopathology, high‐throughput sequencing, bioinformatics analysis and molecular biology methods were used to investigate whether H_2_S donor NaHS could alleviate heat stress‐induced testicular injury and to explore the underlying mode of action of H_2_S. Heat stress reduced testis index, sperm quality, sex hormone levels and disturbed testicular structures. High‐throughput transcriptome sequencing and Gene Ontology (GO) enrichment analyses showed that genes involved in inflammation were upregulated in the heat stress group compared to the control group. H_2_S treatment increased testis index, sperm quality and attenuated pathological alternation and hormone disorder in rats exposed to heat stress. Furthermore, H_2_S administration reversed increased inflammatory factors tumor necrosis factor‐α, interleukin‐1β and interleukin‐6, and resulted in a significant decrease in malondialdehyde levels and in increased activity of catalase and ratio of reduced/oxidized glutathione compared to the heat stress group. This study suggests that H_2_S donor NaHS can effectively restore testicular damage caused by heat stress in rats by inhibiting inflammation and oxidative stress.

## Graphical abstract

Heat stress can cause testicular damage and affect fertility. We investigated the potential protective role of hydrogen sulfide (H_2_S) against heat stress‐induced testicular injury and observed that H_2_S donor NaHS can effectively restore testicular damage in rats by inhibiting inflammation and oxidative stress. Our results suggest that H_2_S might be used as a potential therapeutic strategy for heat‐induced testicular injury.

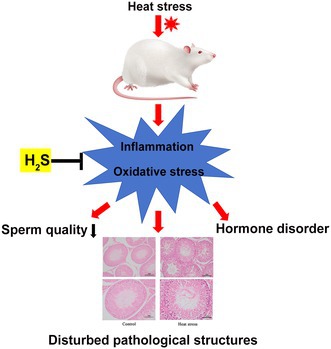

**Abbreviations**
CAT: catalase
CBS: cystathionine β‐synthase
CSE: cystathionine γ‐lyase
DEG: differentially expressed gene
FSH: follicle‐stimulating hormone
GnRH: gonadotrophin‐releasing hormone
GO: Gene Ontology
GSEA: gene set enrichment analysis
GSH/GSSH: ratio of reduced/oxidized glutathione
H&E: hematoxylin and eosin
H_2_S: hydrogen sulfide
IL: interleukin
KEGG: Kyoto Encyclopedia of Genes and Genomes
LH: luteinizing hormone
MDA: malondialdehyde
MSigDB: Molecular Signatures Database
NP: non‐progressive motility rate
PR: progressive motility rate
SD: Sprague–Dawley
TNF: tumor necrosis factor

In mammalian testis, normal spermatogenesis needs a temperature approximately 2–6 °C lower than that of core body. Heat stress has been demonstrated to be significant treats to male reproductive health, particularly by impairing testicular function and sperm quality [1]. Elevated temperature can disrupt the spermatogenic cycle, leading to apoptosis of germ cells, impaired sperm quality and disrupted somatic cell function. Additionally, oxidative stress, inflammation and disruption of the blood–testis barrier integrity have been implicated in the pathogenesis of heat stress‐induced testicular injury [2, 3].

H_2_S, recognized as the third gaseous signaling molecule alongside nitric oxide and carbon monoxide, plays pivotal roles in regulating various physiological processes. Endogenously produced by the enzymes cystathionine β‐synthase (CBS) and cystathionine γ‐lyase (CSE), H_2_S is involved in key functions such as vasodilation, insulin secretion and neurotransmission [4]. Recent research has unveiled its multifaceted roles in various physiological and pathological processes, particularly its anti‐inflammatory and antioxidant properties [5, 6, 7]. However, the role of H_2_S in thermal damage to normal tissue has less been reported.

H_2_S generating enzymes, CBS and CSE, are expressed in testicular tissue of rodents [8, 9]. H_2_S levels are determined in human seminal plasma, and are associated with sperm motility [10]. Several studies have demonstrated that H₂S plays a protective role in the testis against ischemia–reperfusion injury, varicocele (testicular torsion) and exposure to reproductive‐toxic substances [11, 12, 13]. This protection is achieved by inhibiting inflammation, oxidative stress and apoptosis. The expression of CBS and CSE and H_2_S production in the testis of mice were decreased with heat exposure (30 min·day^−1^ at 42 °C for 3 days) [9]. However, the protective role of H_2_S on testis tissue, sperm motility and sex hormone levels have not been demonstrated.

In the present study, by employing a testicular heat stress rat model, we found the occurrence of testicular damage response to heat stress. Meanwhile, the treatment of H_2_S donor NaHS played a preventive measure for the heat‐induced testicular damage. For the first time, we have demonstrated that H_2_S can protect the testis against heat stress in rats.

## Materials and methods

### Animals

Sprague–Dawley (SD) rats were purchased from the Southern Medical University (Guangzhou, China). All animals were housed under controlled conditions, maintained under a 12 h light/dark photocycle at 22 ± 2 °C and 50 ± 10% relative humidity, and were given a standard diet and water *ad libitum*. After the experiments, all rats were sacrificed by exsanguination under anesthesia. All animal procedures were conducted in accordance with the Animal Care Guidelines and with the approval of the Ethical Committee of Institutional Animal Care and Use at General Hospital of Southern Theater Command of PLA (Approval No.: SYDWJJ2022041).

### Experimental design

Two sets of experiments were conducted in the present study. In the first set, 15 male SD rats aged 10–12 weeks with a body weight in the range 220–250 g were randomly divided into three groups: control, heat stress for 7 days and heat stress for 14 days. Rats in the control group were maintained at 22 ± 2 °C and 50 ± 10% relative humidity. In the heat stress group, rats were exposed intermittently to high temperature: 38 ± 5 °C and 80 ± 5% relative humidity fprt 40 min·day^−1^ for 7 and 14 days, respectively. In the second set of experiments, 20 male SD rats were randomly divided into four groups: control, heat stress for 14 days, heat stress for 14 days with NaHS treatment and NaHS treatment alone. The rats were treated with NaHS for 14 days, and the dosages was based on previous reports [14]. Subsequently, the rats were euthanized by exsanguination under anesthesia, and blood and tissues were subsequently collected.

### Measurement of catalase (CAT) activity, reduced and oxidized glutathione content (GSH/GSSG) and malondialdehyde (MDA) levels

One hundred and fifty milligram of testis tissues were homogenized in cold phosphate‐buffered saline and centrifuged at 1500 ***g*** for 15 min and the supernatant was collected. The ratio of GSH/GSSG and CAT activity in the testicular tissues were measured using a GSH/GSSG colorimetric assay kit and CAT activity assay kit, respectively. All kits were purchased from Nanjing Jiancheng Biotechnology Co Ltd (Jiangsu, China). For measurement of MDA levels, 100 mg of testicular tissues was homogenized in 1 mL of 1.15% KCl solution containing 0.85% NaCl. Homogenates were then centrifuged at 1500 ***g*** for 15 min, and the supernatant was collected. The levels of MDA were determined using an MDA assay (Nanjing Jiancheng Biotechnology Co Ltd).

### Histological analysis and organ index calculations

The rats were sacrificed, and testicular tissues (one side) were fixed in Bouin's solution at 4 °C for 48 h. The fixed samples were then embedded in paraffin and sectioned into 4‐mm thick slices. These sections were stained with hematoxylin and eosin (H&E) and testicular injury was evaluated using Johnson's scoring method.

The organ index, representing the ratio of organ weight to body weight, was calculated by dividing the wet mass of the organ by the body weight of the rat.

### Sperm motility and viability analysis

Spermatozoa were isolated from the epididymis of rats. Briefly, the epididymal tissues were minced into pieces in 1 mL of phosphate‐buffered saline to release the sperm. The suspension was incubated at 37 °C for 30 min. Subsequently, 10 μL of the sperm suspension was placed on a counting chamber, and sperm motility was assessed under light microscopy at 40× magnification by counting 200 sperm cells. Sperm viability was determined using the eosin‐nigrosin staining technique. For this, 20 μL of the sperm suspension was mixed with 20 μL of staining solution, and the mixture was spread onto a glass slide. The sample was observed under light microscopy at 40× magnification. Viable sperm cells remained colorless, whereas non‐viable sperm cells were stained red. In total, 200 sperm cells were examined for each sample, and the percentage of stained (non‐viable) sperm was calculated. Each sample was evaluated in duplicate by two technicians, and the results were averaged after error analysis to ensure accuracy.

### Measurement of hormone levels

To measure the testosterone, follicle‐stimulating hormone (FSH), luteinizing hormone (LH) and gonadotrophin‐releasing hormone (GnRH) levels, blood was centrifuged at 1000 ***g*** for 15 min, and then the serum levels of hormone were was assayed using commercial ELISA kits (CUSABIO, Wuhan, China) in accordance with the manufacturer's instructions.

### Measurement of interleukin (IL)‐1β, IL‐6 and tumor necrosis factor (TNF)‐α levels

The concentrations of IL‐1β, IL‐6 and TNF‐α in testis tissues were determined using commercial ELISA kits (CUSABIO) in accordance with the manufacturer's instructions.

### Transcriptome sequencing analysis

RNA‐sequencing was conducted by Novogene Co., Ltd (Beijing, China). Briefly, the fragments per kilobase of exon model per million mapped fragments of each gene were calculated. The expression profiles of the differentially expressed genes (DEGs) were obtained using the R package deseq2 (https://bioconductor.org/packages/release/bioc/html/DESeq2.html). DEGs were identified based on the criteria of *P* < 0.05 and |log_2_ fold‐change| ≥ 0.585. To visualize the expression patterns, we created an expression heatmap using the R package pheatmap (https://github.com/raivokolde/pheatmap) and a volcano plot using the R package ggplot2 (https://github.com/tidyverse/ggplot2). Additionally, DEGs were analyzed for enrichment using Gene Ontology (GO) (http://geneontology.org) and Kyoto Encyclopedia of Genes and Genomes (KEGG) (https://www.genome.jp/kegg) with a significance threshold defined as *P* < 0.05. Gene set enrichment analysis (GSEA) was conducted using the R package clusterprofiler (https://github.com/YuLab‐SMU/clusterProfiler). The predefined gene sets included the GO gene sets, the KEGG gene sets and the HALLMARK pathway gene sets, all of which were sourced from the Molecular Signatures Database (MSigDB) (https://www.gsea‐msigdb.org/gsea/msigdb/index.jsp). Gene sets with an absolute normalized enrichment score > 1, *P* < 0.05 and false discovery rate < 0.25 were considered significantly enriched.

### Statistical analysis

Statistical analyses were conducted using spss, version 21 (IBM Corp., Armonk, NY, USA). Data are presented as the mean ± SEM. The Shapiro–Wilk test was used to assess the normality of the data distribution. Statistical significance was determined based on sample distribution and variance homogeneity. Multigroup comparisons used one‐way and two‐way ANOVA with Bonferroni's post‐hoc test. *P* < 0.05 was considered statistically significant.

## Results

### Heat stress reduced testis index, sperm quality and disturbed pathological structures and hormone levels in the rats

**Fig. 1:**
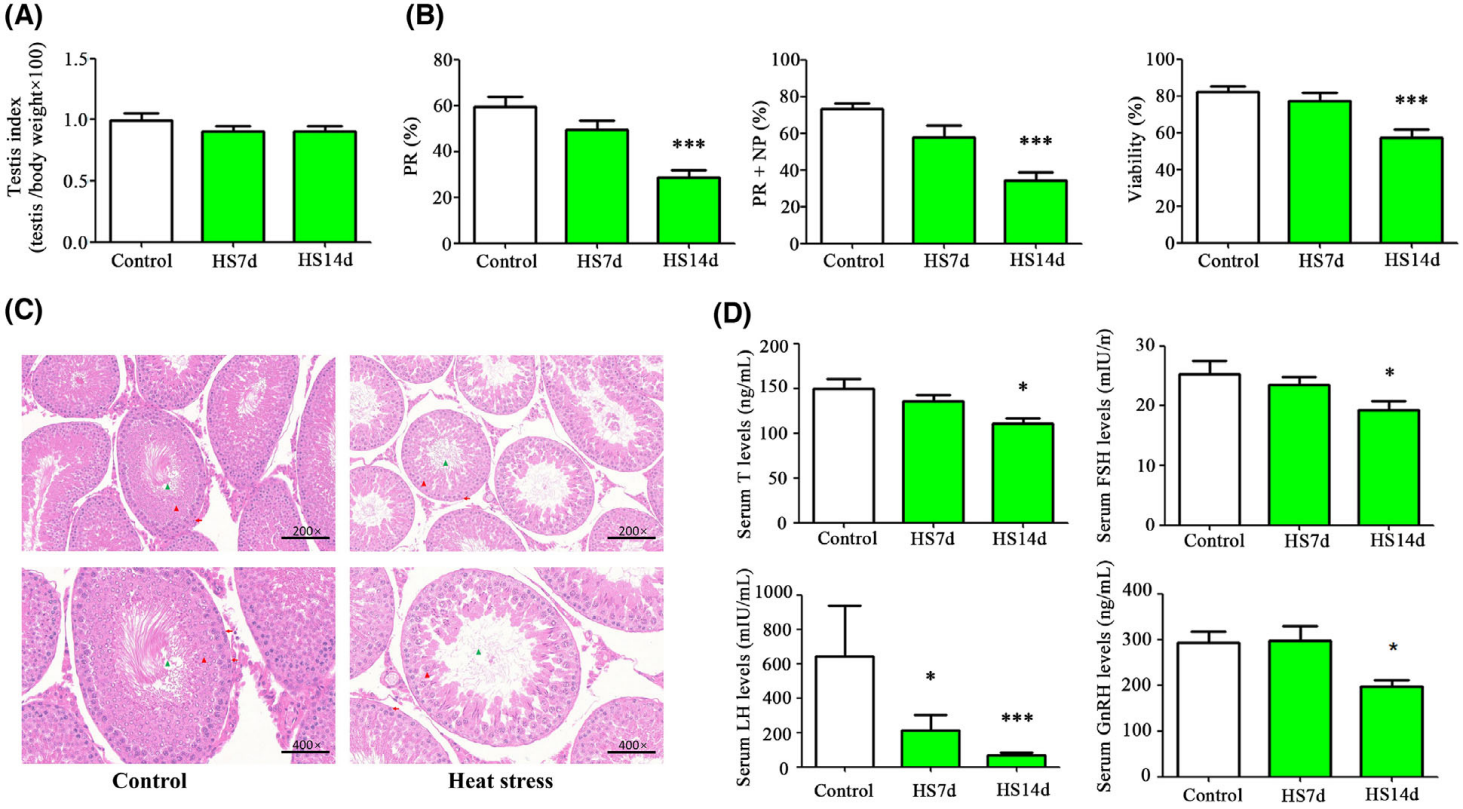
Heat stress reduced testis index, sperm quality, hormone levels and disrupted photography of testes. (A) Testicular organ index. (B) Sperm motility and viability. (C) H&E staining and pathology of testes. (D) Testosterone, FSH, LH and GnRH levels. FSH, follicle‐stimulating hormone; GnRH, gonadotrophin releasing hormone; H&E staining, hematoxylin–eosin staining; HS14d, heat stress for 14 days; HS7d, heat stress for 7 days; LH, luteinizing hormone; NP, non‐progressive motility; PR, progressive motility; T, testosterone. Values are reported as the mean ± SEM (*n* = 5). Statistical analysis was performed by one‐way ANOVA followed by Bonferroni's post‐hoc test. *P < 0.05, ***P < 0.001 versus the control group. Red arrows, spermatogonium; red triangle, spermatocyte; green triangle, sperm.

In this study, rats in the heat stress group were maintained at 38 ± 0.5 °C and 80 ± 5% relative humidity for 40 min per day for 7 and 14 days. By contrast, the rats in the control group were kept at 22 ± 2 °C and 50 ± 10% humidity. After 14 days of heat exposure, progressive motility (PR) decreased by 51.7% compared to the control group. PR + NP (non‐progressive motility) decreased by 53.3% compared to the control group. Sperm viability decreased by 30.5% compared to the control group. However, the testis index did not exhibit significant changes (Fig. 1A,B). Exposure to heat stress for 14 days caused damaged spermatogenesis, including reduced spermatogonia, spermatocytes and sperm (Fig. 1C). The levels of sex hormones, including testosterone, FSH, LH and GnRH, were decreased after 14 days of heat exposure (Fig. 1D). LH levels showed a 10‐fold decrease compared to the control group. Testosterone levels decreased by 25.6% compared to the control group. FSH decreased by 23.9% compared to the control group. GnRH decreased by 32.9% compared to the control group.

### RNA‐sequencing results showed that heat stress induced inflammation and oxidative stress in testicular tissue

**Fig. 2:**
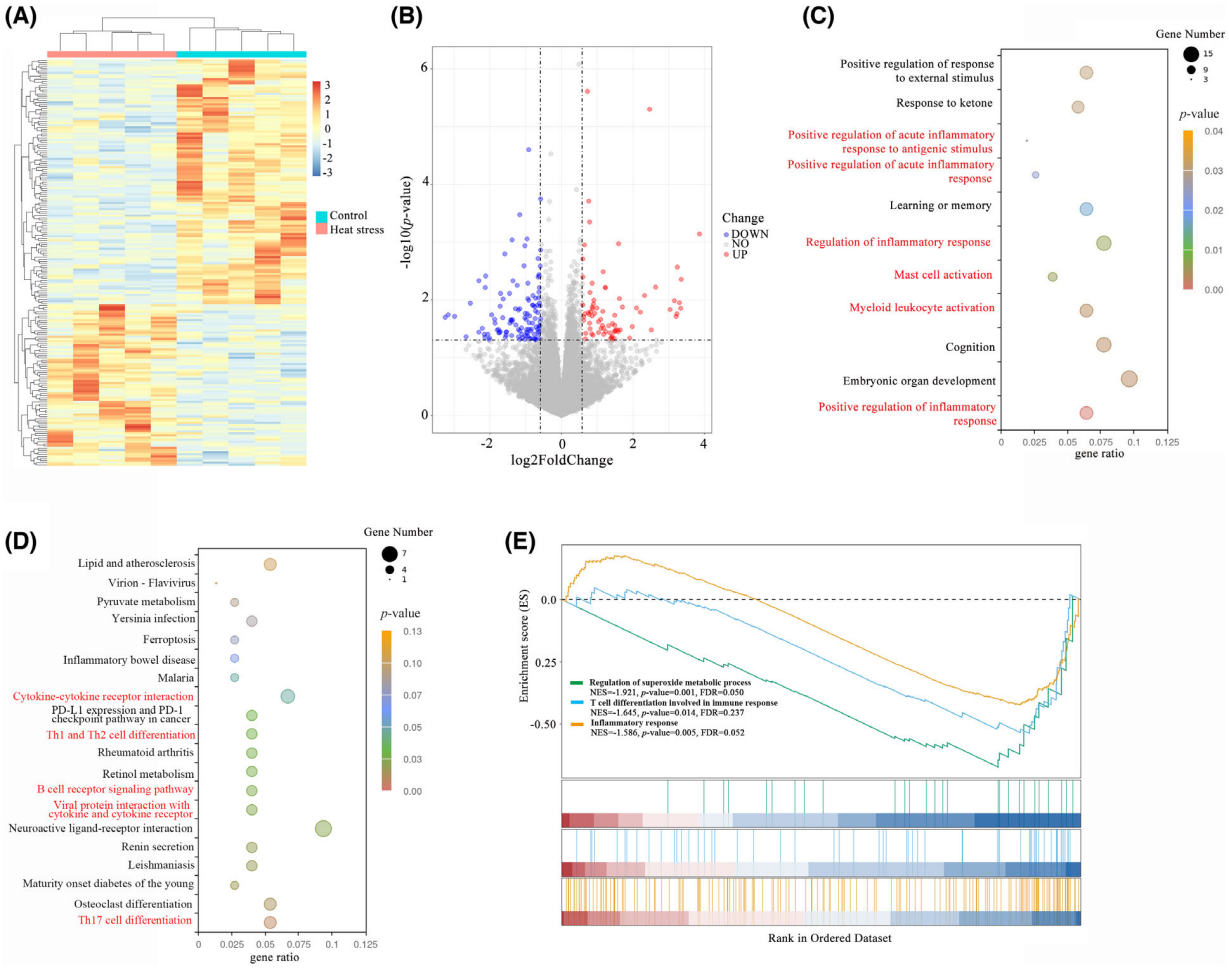
The effects of heat stress on gene expression in testicular tissue (*n* = 5 per group). (A) Heatmap of DEGs between control and heat stress group. (B) Volcano plot of DEGs between control and heat stress group. (C) GO enrichment analysis of DEGs between control and heat stress group. (D) KEGG enrichment analysis of DEGs between control and heat stress group. (E) GSEA revealed significant enrichment of gene sets related to inflammatory responses and regulation of superoxide metabolic processes. DEGs, differentially expressed genes; GO, Gene Ontology; GSEA, gene set enrichment analysis; KEGG, Kyoto Encyclopedia of Genes and Genomes.

Heatmaps and volcano plots were utilized to illustrate the distribution of differentially expressed genes across the groups (Fig. 2A,B). In comparison with the control group, the heat stress group exhibited 93 upregulated and 137 downregulated genes. GO enrichment analyses showed gene sets such as positive regulation of acute inflammatory response to antigenic stimulus, positive regulation of acute inflammatory response, regulation of inflammatory response, mast cell activation, myeloid leukocyte activation and positive regulation of inflammatory response. KEGG enrichment analyses showed gene sets such as cytokine–cytokine receptor interaction, Th1 and Th2 cell differentiation, B cell receptor signaling pathway, viral protein interaction with cytokine and cytokine receptor, and Th17 cell differentiation (Fig. 2C,D). Furthermore, GSEA identified gene sets including inflammatory response, T cell differentiation involved in immune response, and regulation of superoxide metabolic processes were enriched (Fig. 2E). It can be inferred that heat stress may influence testicular tissue at multiple levels, encompassing inflammation and regulation of oxidative stress.

### H_2_S treatment increased testis index, sperm quality and attenuates pathological alternation and hormone disorder in the rats with heat stress

**Fig. 3:**
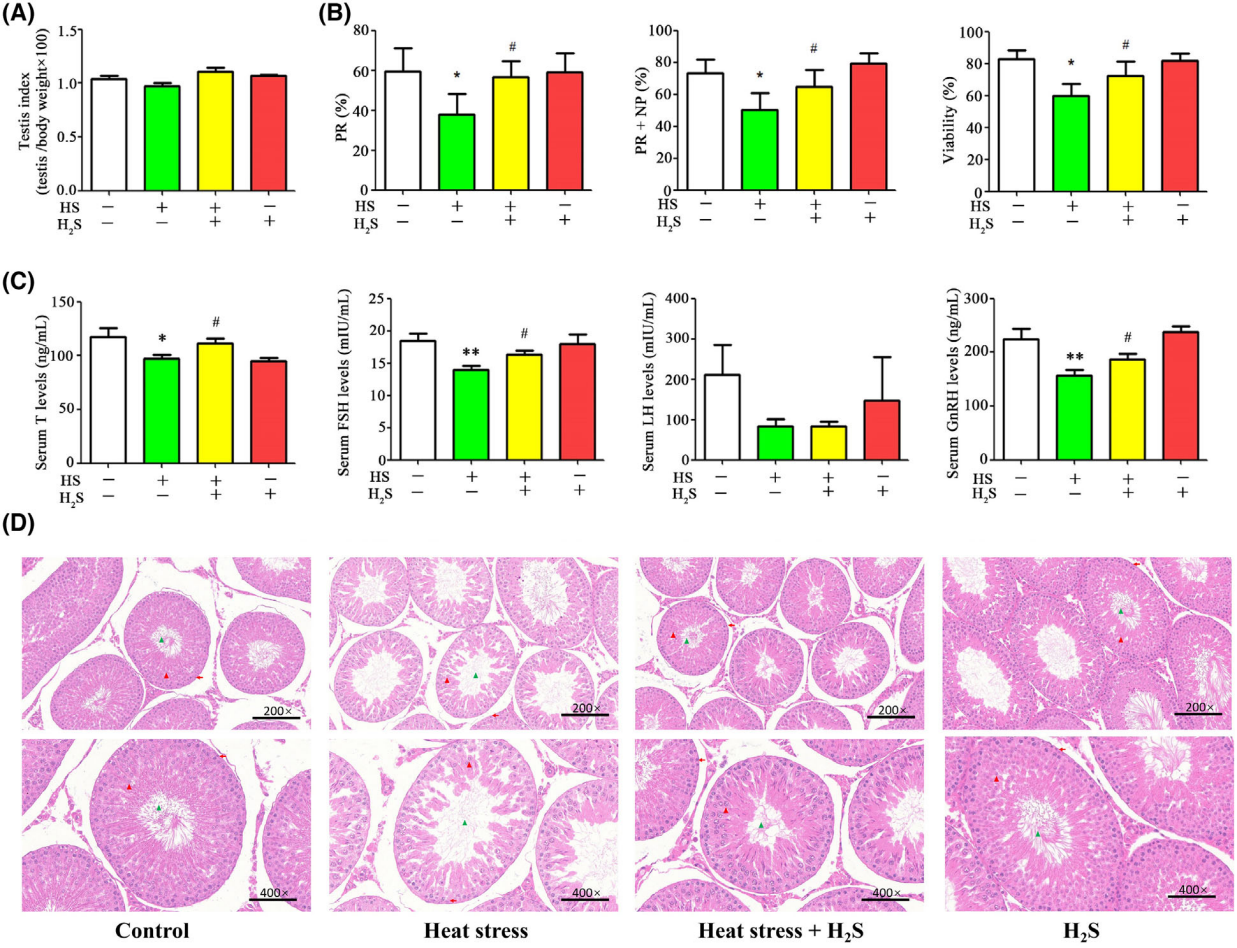
H_2_S treatment increases testis index, sperm quality and attenuates pathological alternation in the testis tissue with heat stress. (A) Testicular organ index. (B) Sperm motility and viability. (C) Testosterone, FSH, LH and GnRH levels. (D) H&E staining and pathology of testes. FSH, follicle‐stimulating hormone; GnRH, gonadotrophin releasing hormone; HS, heat stress; LH, luteinizing hormone; NP, non‐progressive motility; PR, progressive motility; T, testosterone. Values are reported as the mean ± SEM (*n* = 5). Statistical analysis was performed by two‐way ANOVA followed by Bonferroni's post‐hoc test. *P < 0.05, **P < 0.01 versus the control group; ^#^ *P* < 0.05 versus the HS group. Red arrows, spermatogonium; red triangle, spermatocyte; green triangle, sperm.

Prior studies have demonstrated that H_2_S generating enzymes (CBS and CSE) and H_2_S production in testis were significantly reduced in response to heat stress. We assessed the effects of H_2_S donor NaHS on testis index, sperm quality, pathological alternation and hormone levels in testicular tissues of the rats upon to heat insult. There were no significant changes in the testis index following H_2_S treatment (Fig. 3A). However, sperm motility and viability were notably higher in rats subjected to heat stress combined with H_2_S treatment compared to those experiencing heat stress alone (Fig. 3B). Specifically, H_2_S treatment led to a 49.1% increase in sperm progressive motility, a 29.0% increase in PR + NP and a 20.4% improvement in sperm viability compared to the heat stress group. After treatment with H_2_S, the levels of testosterone, FSH and GnRH but not LH in the peripheral blood of rats were significantly increased compared to the heat stress group (Fig. 3C). Testosterone levels increased by 14.6% compared to the heat stress group. FSH increased by 16.2% compared to the heat stress group. GnRH increased by 18.7% compared to the heat stress group. Histological analysis showed that H_2_S treatment significantly promoted spermatogenesis (Fig. 3D). Meanwhile, treatment with H_2_S alone did not impact hormone levels or testis function.

### H_2_S treatment attenuates inflammation and oxidative stress in the rats with heat stress

**Fig. 4:**
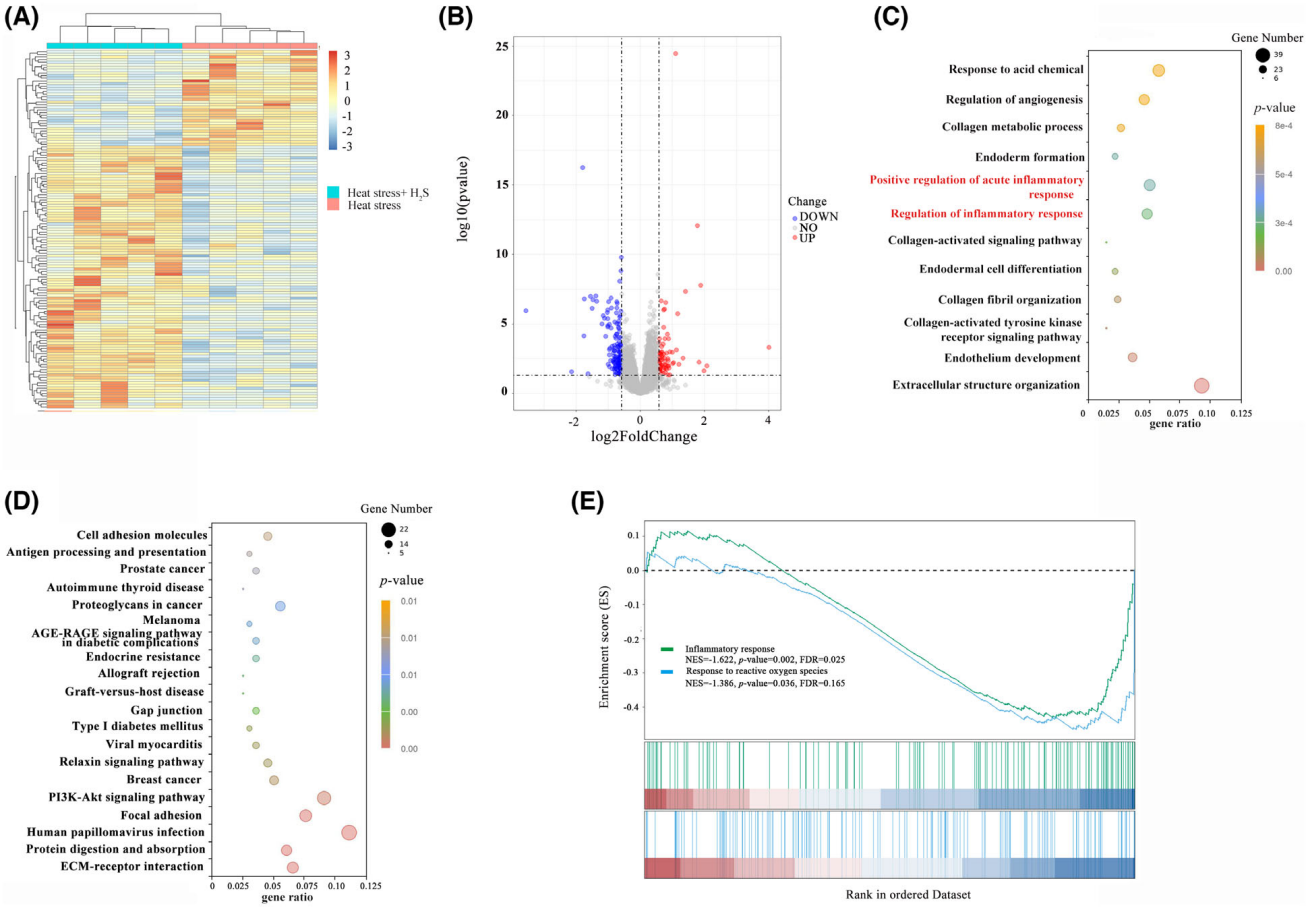
Effect of H_2_S on gene expression in testicular tissue under heat stress (*n* = 5 per group). (A) Heatmap of DEGs between heat stress and heat stress with H_2_S treatment. (B) Volcano plot of DEGs between heat stress and heat stress with H_2_S treatment. (C) GO enrichment analysis of DEGs between heat stress and heat stress with H_2_S treatment. (D) KEGG enrichment of DEGs between heat stress and heat stress with H_2_S treatment. (E) GSEA revealed significant enrichment of gene sets related to inflammatory responses and response to reactive oxygen species. DEGs, differentially expressed genes; GO, gene ontology; GSEA, Gene set enrichment analysis; KEGG, Kyoto Encyclopedia of Genes and Genomes.

To better understand the testicular molecular network affected by H_2_S treatment, RNA‐sequencing was performed. The cluster heatmap showed a significant difference in transcriptome characteristics between the heat stress and H_2_S treatment group (Fig. 4A). The H_2_S treatment group presented 219 upregulated genes and 381 downregulated genes compared to the control group (Fig. 4B). GO enrichment highlighted the pathways including positive regulation of acute inflammatory response, regulation of inflammatory response, collagen metabolic process and extracellular structure organization, etc. KEGG enrichment highlighted the pathways including cell adhesion molecules, gap junction, extracellular matrix‐receptor interaction, phosphoinositide 3‐kinase‐Akt signaling pathway and relaxin signaling pathway, etc. (Fig. 4C,D). GSEA indicated that gene sets encompassing inflammatory response, and response to reactive oxygen species were enriched (Fig. 4E). These results implied that H_2_S treatment may alleviated inflammation and oxidative stress in the testicular tissue.

**Fig. 5:**
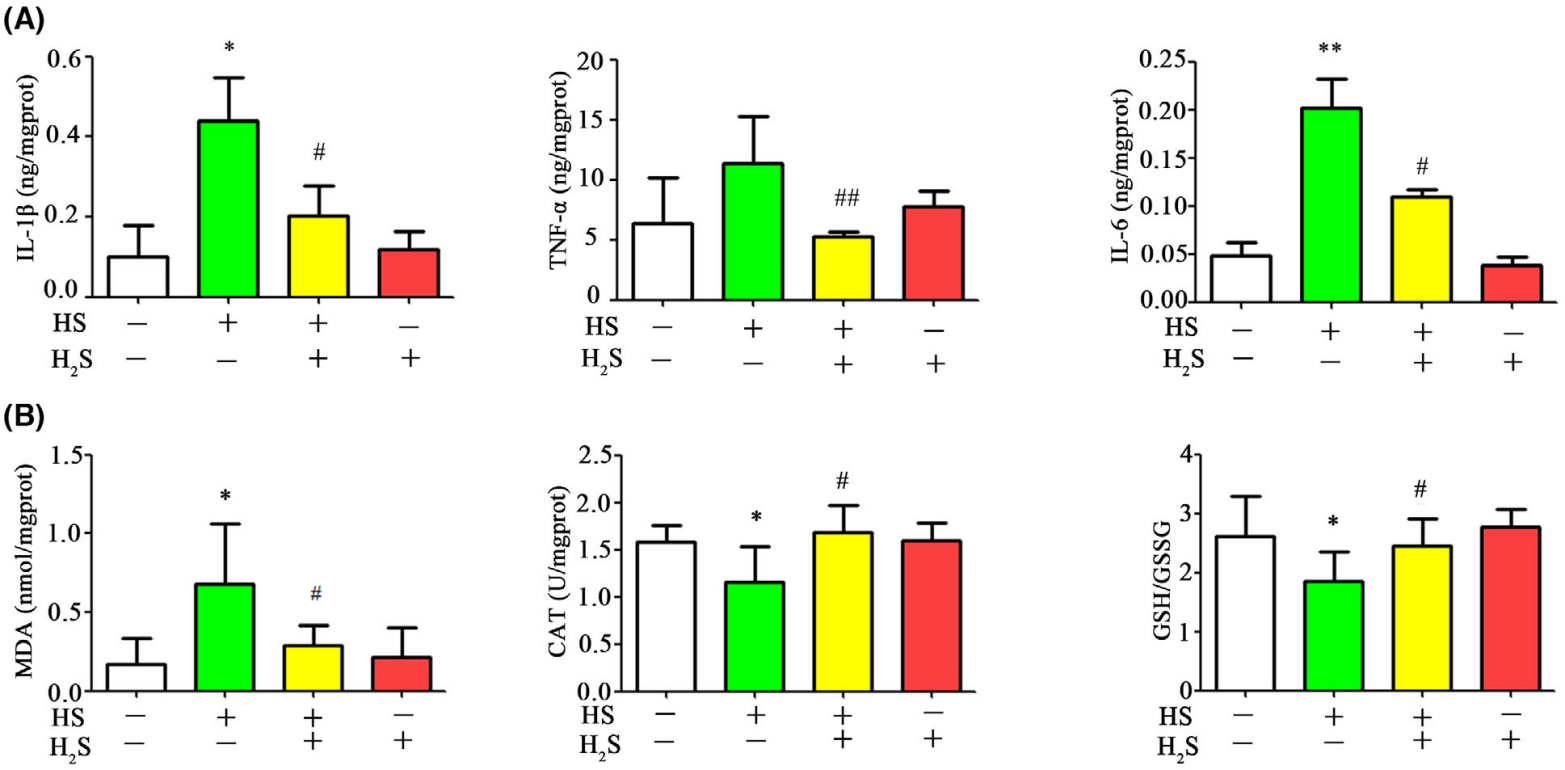
H_2_S donor NaHS treatment reduces the levels of proinflammatory cytokines and oxidative stress in testicular tissues with heat stress. (A) IL‐6, IL‐1β and TNF‐α levels in testicular tissues. (B) MDA, CAT and GSH/GSSG levels in testicular tissues. CAT, catalase; GSH/GSSG, ratio of reduced/oxidized glutathione; HS, heat stress; MDA, malondialdehyde. Values are reported as the mean ± SEM. Statistical analysis was performed by two‐way ANOVA followed by Bonferroni's post‐hoc test. *P < 0.05 versus the control group, **P < 0.01 versus the control group; ^#^ *P* < 0.05, ^##^ *P* < 0.01 versus the HS group.

The levels of inflammation were in the testicular tissue of each group of rats were assessed. After treatment with H_2_S, the inflammatory factors TNF‐α, IL‐1β and IL‐6 were increased by two‐fold (Fig. 5A). Meanwhile, H_2_S treatment resulted in a significant reduction in MDA levels (by 50%) and an increase in CAT activity (1.4‐fold), as well as elevated levels of GSH/GSSH levels (1.3‐fold), compared to the heat stress group (Fig. 5B).

## Discussion

In the present study, we have demonstrated that H_2_S is an important factor in maintaining the physiology function of testes during chronic heat stress. These protective effects are associated with H_2_S physiology function of anti‐inflammatory and antioxidant properties.

Excessive heat exposure to testes leads to a deterioration in sperm quality and impairment of somatic cells [2, 9]. In previous studies, the animal testis was immersed in hot water to establish a testicular heat stress model, which did not match the reality [12, 15]. In the present study, we established a heat stress model through exposing intermittently rats to high temperature and high humidity environment (38 ± 0.5 °C, 80 ± 5% relative humidity). There are important physiological and endocrinological changes that could have indirect effects on the testis in the whole‐body heating model. Serum GnRH levels were decreased in heat stress group, which indicated heat stress‐induced damage to testes is not only associated with physiological changes in testicular tissue, but also with disturbances of pituitary hormones.

In this study, we revealed that whole‐body heat exposure induced oxidative stress and inflammation in testicular tissues. Heat stress decreased the activity of the testicular antioxidant system and disturbed the balance between reactive oxygen species and antioxidants [16]. Then oxidative stress could furtherly activate transcription factors such as nuclear factor kB, which is involved in testicular inflammation and male infertility. Both oxidative stress and inflammation are orchestrated to accentuate each other and induce progressive damage. H_2_S plays an important biological role in inflammatory and oxidative stress processes [6, 17]. Several studies showed that H_2_S can protect testis from inflammation, oxidative stress and apoptosis induced by lipopolysaccharide, varicocele, cisplatin, and ischemia–reperfusion injury [11, 12, 13]. In the present study, we revealed that inflammation and redox imbalance induced by heat stress were reversed by administration of H_2_S donor NaHS.

Testosterone is indispensable for the process of spermatogenesis. Heat stress caused by whole‐body heating or local testicular heating model can lead to the decrease of testosterone secretion [18, 19, 20]. Heat stress significantly decreased endogenous H_2_S production and decreased the expression of CBS and CSE in testes [9]. Meanwhile, H_2_S has been demonstrated to recover testosterone synthesis through affecting the sulfhydrylation of phosphodiesterase [21]. Decrease of H_2_S might be an important cause of insufficient testosterone secretion in heat stress induced testicular tissue.

H_2_S has been demonstrated to play both positive and negative effects *in vitro* and *in vivo* depending on the concentration of H_2_S donors. Zhao *et al*. [22] showed a much higher than physiological concentration of H_2_S declined the motility of mouse spermatozoa *in vivo*. In the present study, 5 mg·kg^−1^ NaHS was used to treat the rats suffered from heat stress. The concentration of NaHS was similar to those in precious reports [14], which ensured that seminal H_2_S was maintained at a relatively stable physiological concentration. Furthermore, in our precious study, H_2_S donor GYY4137 in physiological concentration (10 μm) was used to ameliorate H_2_O_2_‐induced human sperm damage [23], but 100 μm Na_2_S reduced the motility of boar spermatozoa *in vitro* [22].

The present study investigated the protective role of H_2_S against heat stress‐induced testicular insult. Molecular biology techniques and high‐throughput sequencing were employed to assess the impact of heat stress on testicular morphology, testosterone hormone secretion and testicular transcriptome. The results demonstrated that heat stress significantly reduced testis index, sperm quality and disturbed pathological structures and hormone levels. High‐throughput transcriptome sequencing revealed that key differentially expressed genes were clustered in the inflammation between the heat stress group and the control group. H_2_S played a protective role against heat stress‐induced testicular insult through anti‐inflammatory and antioxidant properties. This study sheds new light on the use of H_2_S donor NaHS as a potential therapeutic strategy for treating heat‐induced testicular injury.

## Conflicts of interest

The authors declare that they have no conflicts of interest.

### Peer review

The peer review history for this article is available at https://www.webofscience.com/api/gateway/wos/peer‐review/10.1002/2211‐5463.70010.

## Author contributions

SC and YH developed the concept and designed the study. XG, YY and SG performed the measurement. YH, XC and SX analyzed the data and interpreted the results. YH and YY drafted the manuscript. All authors critically reviewed the manuscript and approved the final version of the manuscript submitted for publication.

## Data Availability

The data that support the findings of this study are available from the corresponding author upon reasonable request.

## References

[1] Nowicka‐Bauer K and Nixon B (2020) Molecular changes induced by oxidative stress that impair human sperm motility. Antioxidants (Basel) 9, 134. 32033035 10.3390/antiox9020134 PMC7070831

[2] Houston BJ , Nixon B , Martin JH , De Iuliis GN , Trigg NA , Bromfield EG , McEwan KE and Aitken RJ (2018) Heat exposure induces oxidative stress and DNA damage in the male germ line. Biol Reprod 98, 593–606. 29351587 10.1093/biolre/ioy009

[3] O'Flaherty C and Scarlata E (2022) Oxidative stress and reproductive function: the protection of mammalian spermatozoa against oxidative stress. Reproduction 164, F67–F78. 37021966 10.1530/REP-22-0200

[4] Cirino G , Szabo C and Papapetropoulos A (2023) Physiological roles of hydrogen sulfide in mammalian cells, tissues, and organs. Physiol Rev 103, 31–276. 35435014 10.1152/physrev.00028.2021

[5] Bao P , Gong Y , Wang Y , Xu M , Qian Z , Ni X and Lu J (2023) Hydrogen sulfide prevents LPS‐induced depression‐like behavior through the suppression of NLRP3 inflammasome and pyroptosis and the improvement of mitochondrial function in the hippocampus of mice. Biology (Basel) 12, 1092. 37626978 10.3390/biology12081092 PMC10451782

[6] Huang Y , Wang G , Zhou Z , Tang Z , Zhang N , Zhu X and Ni X (2021) Endogenous hydrogen sulfide is an important factor in maintaining arterial oxygen saturation. Front Pharmacol 12, 677110. 34135757 10.3389/fphar.2021.677110 PMC8200772

[7] Zhang N , Zhou Z , Huang Y , Wang G , Tang Z , Lu J , Wang C and Ni X (2023) Reduced hydrogen sulfide production contributes to adrenal insufficiency induced by hypoxia via modulation of NLRP3 inflammasome activation. Redox Rep 28, 2163354. 36661247 10.1080/13510002.2022.2163354 PMC9869992

[8] Jamshidian‐Ghalehsefidi N , Rabiee F , Tavalaee M , Kiani S , Pouriayevali F , Razi M , Dattilo M and Nasr‐Esfahani MH (2023) The role of the transsulfuration pathway in spermatogenesis of vitamin D deficient mice. Sci Rep 13, 19173. 37932339 10.1038/s41598-023-45986-4 PMC10628119

[9] Li G , Xie ZZ , Chua JM , Wong PC and Bian J (2015) Hydrogen sulfide protects testicular germ cells against heat‐induced injury. Nitric Oxide 46, 165–171. 25446250 10.1016/j.niox.2014.10.005

[10] Wang J , Wang W , Li S , Han Y , Zhang P , Meng G , Xiao Y , Xie L , Wang X , Sha J *et al*. (2018) Hydrogen sulfide as a potential target in preventing spermatogenic failure and testicular dysfunction. Antioxid Redox Signal 28, 1447–1462. 28537489 10.1089/ars.2016.6968

[11] Yuksel S , Erginel B , Bingul I , Ozluk Y , Karatay H , Aydın F and Keskin E (2022) The effect of hydrogen sulfide on ischemia/reperfusion injury in an experimental testicular torsion model. J Pediatr Urol 18, 16.e1–e7. 10.1016/j.jpurol.2021.11.019 34937685

[12] Lorian K , Kadkhodaee M , Kianian F , Abdi A , Ranjbaran M , Ashabi G and Seifi B (2020) Long‐term NaHS administration reduces oxidative stress and apoptosis in a rat model of left‐side varicocele. Andrologia 52, e13496. 31793716 10.1111/and.13496

[13] Azarbarz N , Shafiei Seifabadi Z , Moaiedi MZ and Mansouri E (2020) Assessment of the effect of sodium hydrogen sulfide (hydrogen sulfide donor) on cisplatin‐induced testicular toxicity in rats. Environ Sci Pollut Res Int 27, 8119–8128. 31900777 10.1007/s11356-019-07266-5

[14] Aboulhoda BE , Rashed LA , Ahmed H , Obaya EMM , Ibrahim W , Alkafass MAL , Abd El‐Aal SA and ShamsEldeen AM (2021) Hydrogen sulfide and mesenchymal stem cells‐extracted microvesicles attenuate LPS‐induced Alzheimer's disease. J Cell Physiol 236, 5994–6010. 33481268 10.1002/jcp.30283

[15] Zhang P , Zheng Y , Lv Y , Li F , Su L , Qin Y and Zeng W (2020) Melatonin protects the mouse testis against heat‐induced damage. Mol Hum Reprod 26, 65–79. 31943111 10.1093/molehr/gaaa002

[16] Slimen IB , Najar T , Ghram A , Dabbebi H , Ben Mrad M and Abdrabbah M (2014) Reactive oxygen species, heat stress and oxidative‐induced mitochondrial damage. A review. Int J Hyperthermia 30, 513–523. 25354680 10.3109/02656736.2014.971446

[17] Zhang HX , Liu SJ , Tang XL , Duan GL , Ni X , Zhu XY , Liu YJ and Wang CN (2016) H2S attenuates LPS‐induced acute lung injury by reducing oxidative/nitrative stress and inflammation. Cell Physiol Biochem 40, 1603–1612. 28006762 10.1159/000453210

[18] Jia X , Li Z , Ren X , Dai P , Li Y and Li C (2020) L‐arginine alleviates the testosterone reduction in heat‐treated mice by upregulating LH secretion, the testicular antioxidant system and expression of steroidogenesis‐related genes. Reprod Fertil Dev 32, 885–892. 32586418 10.1071/RD19303

[19] Jeremy M , Gurusubramanian G , Kharwar RK and Roy VK (2022) Evaluation of a single dose of intra‐testicular insulin treatment in heat‐stressed mice model. Andrologia 54, e14603. 36156807 10.1111/and.14603

[20] Bai L , Zhang Y , Zheng C , Xu S , He Y , Yu G , Huang D , Huang Y , Li M and Xu C (2023) Tanshinone IIA protects mouse testes from heat stress injury by inhibiting apoptosis and TGFβ1/Smad2/Smad3 signaling pathway. Cell Stress Chaperones 28, 749–759. 37610501 10.1007/s12192-023-01367-4 PMC10746600

[21] Wang J , Wang J , Shen T , Hong R , Tang S and Zhao X (2021) H_2_S catalysed by CBS regulates testosterone synthesis through affecting the sulfhydrylation of PDE. J Cell Mol Med 25, 3460–3468. 33713531 10.1111/jcmm.16428 PMC8034449

[22] Zhao Y , Zhang WD , Liu XQ , Zhang PF , Hao YN , Li L , Chen L , Shen W , Tang XF , Min LJ *et al*. (2016) Hydrogen sulfide and/or ammonia reduces spermatozoa motility through AMPK/AKT related pathways. Sci Rep 6, 37884. 27883089 10.1038/srep37884 PMC5121643

[23] Huang Y , Gan R , Zhang M , Lin D , Cheng Y and Guo X (2024) Treatment of human sperm with GYY4137 increases sperm motility and resistance to oxidative stress. Zygote 32, 360–365. 39474802 10.1017/S0967199424000340

